# The Salutary Effects of Catalpol on Diesel Exhaust Particles-Induced Thrombogenic Changes and Cardiac Oxidative Stress, Inflammation and Apoptosis

**DOI:** 10.3390/biomedicines10010099

**Published:** 2022-01-04

**Authors:** Abderrahim Nemmar, Sumaya Beegam, Nur Elena Zaaba, Salem Alblooshi, Saleh Alseiari, Badreldin H. Ali

**Affiliations:** 1 Department of Physiology, College of Medicine and Health Sciences, United Arab Emirates University, Al Ain P.O. Box 17666, United Arab Emirates; sumayab@uaeu.ac.ae (S.B.); elenazaaba@uaeu.ac.ae (N.E.Z.); 201710986@uaeu.ac.ae (S.A.); 201702187@uaeu.ac.ae (S.A.); 2 Department of Pharmacology and Clinical Pharmacy, College of Medicine & Health Sciences, Sultan Qaboos University, P.O. Box 35, Muscat 123, Oman; alibadreldin@hotmail.com

**Keywords:** catalpol, diesel exhaust particles, coagulation, heart inflammation, oxidative stress, apoptosis, DNA damage

## Abstract

Inhaled particulate air pollution exerts pulmonary inflammation and cardiovascular toxicity through secondary systemic effects due to oxidative stress and inflammation. Catalpol, an iridiod glucoside, extracted from the roots of *Rehmannia glutinosa Libosch,* has been reported to possess anti-inflammatory and antioxidant properties. Yet, the potential ameliorative effects of catalpol on particulate air pollution—induced cardiovascular toxicity, has not been studied so far. Hence, we evaluated the possible mitigating mechanism of catalpol (5 mg/kg) which was administered to mice by intraperitoneal injection one hour before the intratracheal (i.t.) administration of a relevant type of pollutant particle, viz. diesel exhaust particles (DEPs, 30 µg/mouse). Twenty-four hours after the lung deposition of DEPs, several cardiovascular endpoints were evaluated. DEPs caused a significant shortening of the thrombotic occlusion time in pial microvessels in vivo, induced platelet aggregation in vitro, and reduced the prothrombin time and the activated partial thromboplastin time. All these actions were effectively mitigated by catalpol pretreatment. Likewise, catalpol inhibited the increase of the plasma concentration of C-reactive proteins, fibrinogen, plasminogen activator inhibitor-1 and P- and E-selectins, induced by DEPs. Moreover, in heart tissue, catalpol inhibited the increase of markers of oxidative (lipid peroxidation and superoxide dismutase) and nitrosative (nitric oxide) stress, and inflammation (tumor necrosis factor α, interleukin (IL)-6 and IL-1β) triggered by lung exposure to DEPs. Exposure to DEPs also caused heart DNA damage and increased the levels of cytochrome C and cleaved caspase, and these effects were significantly diminished by the catalpol pretreatment. Moreover, catalpol significantly reduced the DEPs-induced increase of the nuclear factor κB (NFκB) in the heart. In conclusion, catalpol significantly ameliorated DEPs–induced procoagulant events and heart oxidative and nitrosative stress, inflammation, DNA damage and apoptosis, at least partly, through the inhibition of NFκB activation.

## 1. Introduction

It is well established that ambient particulate air pollution is associated with a significant increase in mortality and morbidity. Outdoor air pollution, resulting from particulate matter with an aerodynamic diameter of <2.5 µm (PM_2.5_) has been categorized as the fifth most important risk factor for all causes of mortality [1]. PM_2.5_ is responsible for 7.6% of total global deaths and 4.2% of global disability-adjusted life-years [1]. Exposure to air pollution is associated with a reduction of life expectancy from 1 to 2 years in highly polluted areas [1,2].

Cardiovascular diseases constitute approximately 57% of the deaths from air pollution [1,2]. Epidemiological, clinical and experimental investigations have identified substantial associations between exposure to particulate air pollution and myocardial infarction, stroke, increase in heart rate variability and blood pressure, vascular dysfunction and the augmented vulnerability of the heart to ischaemic damage and elevated susceptibility for thrombosis [3,4,5].

While substantial improvements in air quality have been achieved in some developed countries, many developing countries still report high levels of particulate air pollution [1]. An indication of a safe level of air pollution has not yet been established. In fact, PM_2.5_ levels below those set by the current air quality limit value by the European Union (<25 μg/m^3^) are still associated with substantial adverse cardiovascular effects [6,7]. Thus, it is essential to apply supplementary measures that decrease the cardiovascular toxicity inherent in exposure to air pollution, including the intake of safe dietary natural phytochemicals with established anti-inflammatory and antioxidative properties. 

In urban locations, diesel exhaust emissions represent a substantial source of particulate air pollution [3,8]. Acute pulmonary exposure to diesel exhaust particles (DEPs) in experimental animals and controlled exposure studies to diesel exhaust involving human subjects have reported endothelial dysfunction, alterations in heart rate variability, thrombogenicity and impairment fibrinolysis through mechanisms involving oxidative stress and inflammation [3,5,9]. 

Catalpol, an iridiod glucoside extracted from the roots of *Rehmannia glutinosa* Libosch, has been utilized in traditional Korean and Chinese medicine to treat numerous disorders, including diabetes mellitus, neuronal disorders, and inflammation [10]. Additionally, various experimental studies have demonstrated that catalpol displays anti-diabetic, nephroprotective, cardioprotective, antioxidant and anti-inflammatory actions [10,11,12]. Therefore, since the mechanisms underlying the adverse cardiovascular effects of DEPs involve oxidative stress and inflammation, we thought it of interest to assess the possible salutary effects of catalpol on DEPs-induced cardiovascular toxicity. As far as we know, this is the first report investigating such an interaction. Our study aims to evaluate the potential protective effects of catalpol on DEPs-induced cardiovascular complication, by assessing various relevant endpoints including thrombosis in vivo and in vitro and cardiac oxidative stress, inflammation, DNA damage, apoptosis and the expression of nuclear factor-κB (NF-κB). 

## 2. Materials and Methods

### 2.1. Diesel Exhaust Particles (DEPs) and Catalpol

The DEPs were procured from the National Institute of Standards and Technology (NIST, Gaithersburg, MD, USA), and were suspended in sterile saline (NaCl 0.9%) with Tween 80 (0.01%). To reduce particle aggregation, DEPs suspensions were sonicated for 20 min, vigorously vortexed prior to suspension, and diluted before intratracheal (i.t.) administration. Control animals were administered with saline containing Tween 80 (0.01%). The DEPs used in this work were previously examined by transmission electron microscopy, which revealed the presence of small aggregates (<100 nm) of carbonaceous particles. The majority of the aggregates were less than 1 μm in diameter [13]. The analysis of the same DEPs from the same source revealed a geometric mean aerodynamic diameter of 215 nm [14].

Catalpol (purity ≥ 98%) was procured using Sigma Chemical (St. Louis, MO, USA). 

### 2.2. Mice Treatments

This project was approved by the Institutional Review Board of the United Arab Emirates University (protocol code ERA_2019_5876, approved on 9 April 2019), and the experiments were ethically carried out in accordance with the approved protocols.

Both male and female BALB/C mice (6 to 8 weeks; College of Medicine and Health Sciences animal house, UAEU) were kept in temperature-controlled (22 ± 1 °C) rooms with a 12 h light cycle and 12 h dark cycle (lights on at 6 00 a.m.). They had unrestricted access to laboratory chow and tap water ad libitum.

Mice were exposed to DEPs by intratracheal (i.t.) instillation [15,16,17,18,19]. To this end, all mice were deeply anesthetized using 5% isoflurane (Surgivet^®^ model 100 vaporizer). Using a 24 G catheter, they were i.t. instilled with either saline or the DEPs suspension (30 μg/animal) [19,20]. Each administration consisted of a volume of 100 μL, followed by a bolus of 0.3 mL air. Catalpol was administered by intraperitoneal injection (5 mg/kg), one hour prior to i.t. administration, to either DEPs or saline. The dose of catalpol used in the present work has been selected from previous studies that demonstrated its effectiveness, including in reducing oxidative stress and exerting cardioprotective effects against an ischemia/reperfusion insult in rats [11], increasing brain angiogenesis and ameliorating the edema of the brain capillary endothelial cells, following permanent middle cerebral artery occlusion in rats [21], ameliorating atherosclerotic lesions in hypercholesterolemic rabbits [22] and improving behavioral impairment and cerebral blood flow in rats after cerebral ischemia [23]. The animals were separated at random into four groups and were treated as follows:

- Group 1: Normal saline administered i.p. 1 h prior the i.t. administration of saline;
- Group 2: Normal saline administered i.p. 1 h prior i.t. administration of DEPs (30 μg/mouse);
- Group 3: Catalpol (5 mg/kg) administered i.p. 1 h prior the i.t. administration of saline;
- Group 4: Catalpol (5 mg/kg) administered i.p. 1 h prior i.t. administration of DEPs (30 μg/mouse).

Twenty-four hours after the pulmonary exposure to either saline or DEPs, various cardiovascular parameters were evaluated. 

Twenty-four hours following the pulmonary exposure to DEPs or the vehicle, various cardiovascular parameters were measured. 

### 2.3. Assessment of Thrombosis in Pial Arterioles and Venules In Vivo

An in vivo evaluation of thrombogenesis in pial arterioles and venules was measured following the i.t. instillation of DEPs or saline with or without catalpol pretreatment, according to a previously reported technique [18,24]. 

### 2.4. Evaluation of Platelet Aggregation in Whole Blood In Vitro

The platelet aggregation in the whole blood was carried out as reported earlier [18,24]. Following anesthesia, blood from mice, that had been i.t. instilled with DEPs or saline and with or without catalpol administration, was collected from the inferior vena cava and placed in citrate (3.2%), and 100-µL samples were added to the well of a Merlin coagulometer MC 1 VET (Merlin, Lemgo, Germany). The blood samples were incubated for 3 min at 37.2 °C with 1 μM of adenosine diphosphate (ADP), and then stirred for another 3 min. After this, 25-μL samples were removed and fixed on ice in 225 mL cellFix. After fixation, using a VET ABX Micros with mouse card (ABX, Montpellier, France), the single platelets were counted. The level of platelet aggregation, evaluated as a reduction in the single platelets counted in the presence of ADP, was quantified in whole blood collected from mice exposed to either DEPs or saline with or without catalpol treatment. 

### 2.5. Prothrombin Time (PT) and Activated Partial Thromboplastin Time (aPTT) Assessment in Plasma In Vitro

Twenty-four hours after i.t. instillation with DEPs or saline, with or without catalpol treatment, animals were anesthetized, and blood was collected from the inferior vena cava and placed in a citrate solution (3.2%) (ratio of blood to anticoagulant: 9:1). The PT was measured on freshly collected platelet-poor plasma with human relipidated recombinant thromboplastin (Recombiplastin; Instrumentation Laboratory, Orangeburg, NY, United States) with a coagulometer (MC 1 VET, Merlin, Lemgo, Germany) [18,24]. Using the latter coagulometer, the aPTT was assessed with the automated aPTT reagent, purchased from bioMerieux (Durham, NC, USA) [18,24].

### 2.6. Measurement of C-Reactive Protein, Fibrinogen, Plasminogen Activator Inhibitor-1, and P- and E-Selectins Concentrations in Plasma

Twenty-four hours after i.t. instillation with DEPs or saline with or without catalpol treatment, the mice were anesthetized using an i.p. injection of sodium pentobarbital (45 mg/kg), after which blood was collected from the inferior vena cava in 4% EDTA and spun at 4 °C for 15 min at 900 g. The plasma samples obtained were kept at −80 °C as they awaited analysis. The plasma concentrations of CRP (GenWay Biotech, Inc., San Diego, CA, USA), fibrinogen (Molecular Innovation, Southfield, MI, USA), PAI-1 (Molecular Innovation, Southfield, MI, USA), and P- and E-selectins (R&D systems, Minneapolis, MN, USA), were measured with ELISA kits.

### 2.7. Measurement of the Levels of Lipid Peroxidation (LPO), Superoxide Dismutase (SOD), Nitric Oxide (NO), Tumor Necrosis Factor α (TNFα), Interleukin 1β (IL1β) and IL-6 in Heart Tissue Homogenates

Heart homogenates preparation for the measurement of markers of oxidative stress and inflammation were prepared as described before [18]. NADPH-dependent membrane lipid peroxidation was measured as a thiobarbituric acid reactive substance, using malondehydehyde as standard (Sigma-Aldrich Fine Chemicals, St Louis, MO, United States). The activity of SOD was quantified as per the vendor’s protocols (Cayman Chemicals, Ann Arbor, MI, United States). The measurement of nitric oxide (NO) was performed with a total NO assay kit (R&D systems, Minneapolis, MN, United States), which was used to quantify the more stable NO metabolites, NO_2_^−^ and NO_3_^−^ [25].

The concentrations of TNFα, IL1β and IL-6 were measured using commercially available kits (Duo Set, R&D systems, Minneapolis, MN, USA). 

### 2.8. Measurement of Cytochrome C, Cleaved Caspase, Phosphorylated NF-κB and Phosphorylated IκBα in Heart Homogenates

The measurement of cytochrome C (R&D Systems, Minneapolis, MN, USA), cleaved caspase C (R&D Systems, Minneapolis, MN, USA) and phosphorylated NF-κB (Cell Signalling Technology, Danvers, MA, USA) in heart homogenates, obtained from mice i.t. instilled with DEPs or saline with or without catalpol treatment, were obtained using commercially available ELISA kits. Moreover, protein expressions for phosphorylated NF-κB and phosphorylated NF-κB inhibitor α (IκBα) were further measured using the Western blotting technique, as previously reported [20,26,27].

### 2.9. DNA Damage

The hearts from mice i.t. instilled with DEPs or saline with or without catalpol treatment were collected immediately after sacrifice and processed for the assessment of DNA damage by COMET assay as reported earlier [28,29]. The assessment of DNA migration, which includes the nucleus diameter and migrated DNA, was evaluated by means of the image analysis Axiovision 3.1 software (Carl Zeiss, Toronto, ON, Canada) as described before [30,31].

### 2.10. Statistical Analysis

A statistical analysis was performed using GraphPad Prism (version 7; GraphPad Software Inc, San Diego, CA, USA). The results are expressed as the mean± SEM. Comparisons among the four studied groups were performed with a one-way analysis of variance followed by Holm-Sidak’s multiple comparisons test. *P* values of < 0.05 were considered to be significantly different.

## 3. Results

### 3.1. Thrombosis in Pial Microvessels

**Figure 1:**
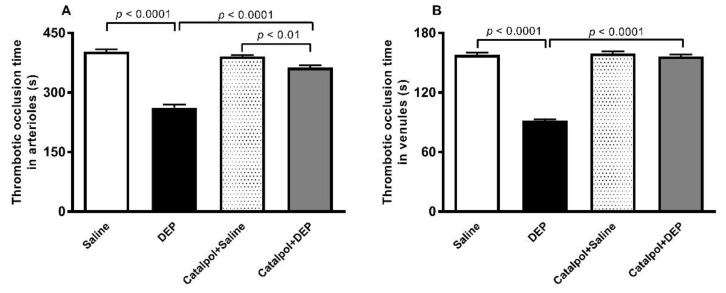
Thrombotic occlusion time in pial arterioles (**A**) and venules (**B**), 24 h after intratracheal instillation of saline or diesel exhaust particles (DEPs; 30 μg/mouse) with or without catalpol (5 mg/kg) pretreatment. Data are mean ± SEM (*n* = 7–10). Statistical analysis by one-way analysis of variance followed by Holm-Sidak’s multiple comparisons test.

Figure 1 shows that, compared with the control group, pulmonary exposure to DEPs caused a significant shortening in the thrombotic occlusion time in pial arterioles (*p* < 0.0001) and venules (*p* < 0.0001). The latter effects were prevented to a significant degree by pretreatment with catalpol (*p* < 0.0001). In pial arterioles, there was a slight but significant difference between the catalpol+saline and catalpol+DEPs groups (*p* < 0.01). However, in pial venules there was no difference in the thrombotic occlusion time between these two groups.

### 3.2. Platelet Aggregation in Whole Blood, PT and aPTT In Vitro

**Figure 2:**
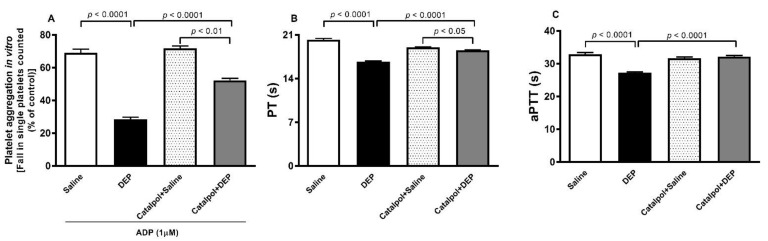
In vitro platelet aggregation in whole blood incubated with ADP (1µM) (**A**). The blood was collected from mice 24 h after intratracheal instillation of saline or diesel exhaust particles (DEPs; 30 μg/mouse) with or without catalpol (5 mg/kg) pretreatment. Platelet aggregation was evaluated by quantifying the fall in single platelets, counted due to aggregation induced by DEPs. The degree of platelet aggregation was expressed as the percentage obtained in untreated whole blood from untreated mice. The activated partial thromboplastin time (aPTT, (**B**)) and prothrombin time (PT, (**C**)) assessed on plasma samples obtained 24 h after intratracheal instillation of saline or diesel exhaust particles (DEPs; 30 μg/mouse) with or without catalpol (5 mg/kg) pretreatment. Data are mean ± SEM (*n* = 6). Statistical analysis by one-way analysis of variance followed by Holm-Sidak’s multiple comparisons test.

As illustrated in Figure 2A, compared with the control group, the addition of ADP (1 µM) to the whole blood collected from mice, i.t. instilled with DEPs, caused platelet aggregation in vitro (*p* < 0.0001). This effect was significantly ameliorated by the pretreatment with catalpol (*p* < 0.0001). There was a significant difference in the platelet aggregation between catalpol+saline and catalpol+DEPs groups (*p* < 0.01).

Likewise, the PT (Figure 2B) and aPTT (Figure 2C) were significantly shortened in the plasma of mice exposed to DEPs (*p* < 0.0001). These effects were significantly reduced following the pretreatment with catalpol. The PT recorded in the catalpol+DEPs group was slightly, but significantly, shorter than that measured in the catalpol+saline (*p* < 0.05). 

### 3.3. CRP, Fibrinogen, PAI-1, and P- and E-Selectins Concentrations in Plasma

**Figure 3:**
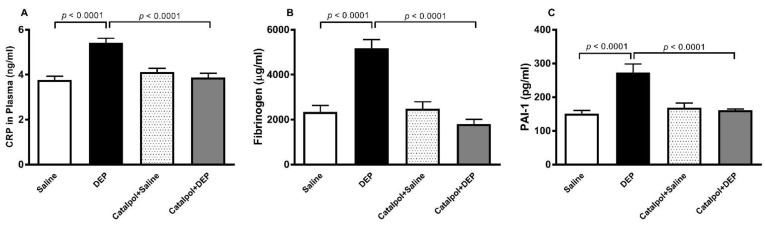
C-reactive protein (**A**), fibrinogen (**B**) and plasminogen activator inhibitor-1 (PAI-1; (**C**)) concentrations in plasma, 24 h after intratracheal instillation of saline or diesel exhaust particles (DEPs; 30 μg/mouse) with or without catalpol (5 mg/kg) pretreatment. Data are mean ± SEM (*n* = 8). Statistical analysis by one-way analysis of variance followed by Holm-Sidak’s multiple comparisons test.

Figure 3 illustrates the plasma concentrations of CRP, fibrinogen, and PAI-1 after i.t. administration of DEPs or saline with or without catalpol pretreatment. The pulmonary exposure to DEPs induced a significant increase in the concentrations of CRP, fibrinogen and PAI-1 (*p* < 0.0001). Pretreatment with catalpol significantly reversed these effects (*p* < 0.0001). 

**Figure 4:**
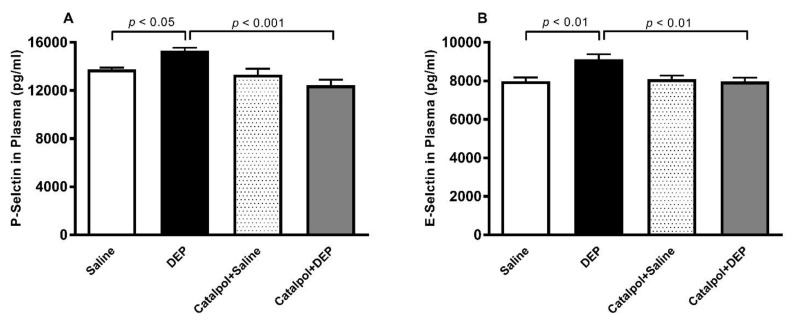
P-selectin (**A**) and E-selectin (**B**) concentrations in plasma, 24 h after intratracheal instillation of saline or diesel exhaust particles (DEPs; 30 μg/mouse) with or without catalpol (5 mg/kg) pretreatment. Data are mean ± SEM (*n* = 7–8). Statistical analysis by one-way analysis of variance followed by Holm-Sidak’s multiple comparisons test.

Figure 4 shows that the i.t. administration of DEPs caused a significant increase in the plasma concentration of P- and E-selectin (*p* < 0.01–0.05). The pretreatment with catalpol almost completely prevented these effects (*p* < 0.001–0.01).

### 3.4. TNFα, IL-1β and IL-6 in Heart Homogenates

**Figure 5:**
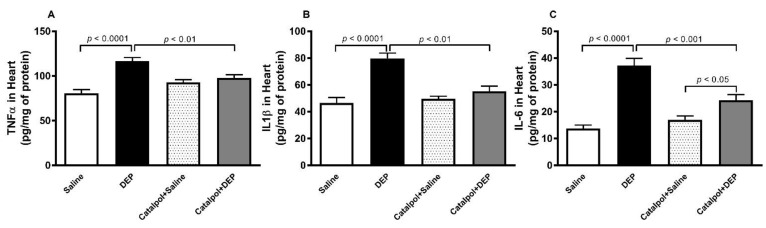
Tumor necrosis factor-α (**A**), interleukin (IL)-1β (**B**) and IL-6 (**C**) concentrations in heart homogenates of mice, 24 h after intratracheal instillation of saline or diesel exhaust particles (DEPs; 30 μg/mouse) with or without catalpol (5 mg/kg) pretreatment. Data are mean ± SEM (*n* = 8). Statistical analysis by one-way analysis of variance followed by Holm-Sidak’s multiple comparisons test.

Figure 5 shows that, compared with the control group, pulmonary exposure to DEPs caused a significant increase in the concentrations of TNFα, IL-1β and IL-6 in heart homogenates (*p* < 0.0001). The latter effects were significantly attenuated by pretreatment with catalpol (*p* < 0.001–0.01). Compared with the catalpol+saline group, the concentration of IL-6 in heart homogenates of the catalpol+DEPs group was slightly but significantly higher (*p* < 0.05).

### 3.5. LPO, SOD and NO in Heart Homogenates

**Figure 6:**
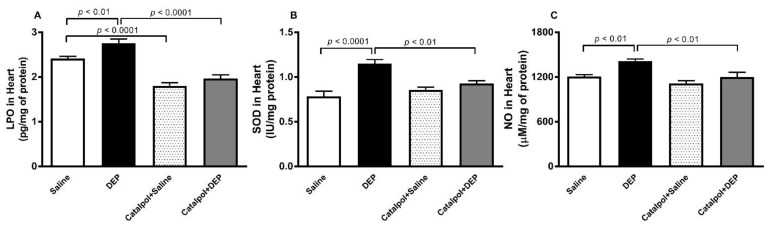
Lipid peroxidation (**A**), superoxide dismutase (SOD) (**B**) and total nitric oxide (NO) (**C**) levels in heart homogenates of mice, 24 h after intratracheal instillation of saline or diesel exhaust particles (DEPs; 30 μg/mouse) with or without catalpol (5 mg/kg) pretreatment. Data are mean ± SEM (*n* = 7–8). Statistical analysis by one-way analysis of variance followed by Holm-Sidak’s multiple comparisons test.

Compared with the control group, the i.t. instillation of DEPs induced a significant increase in the levels of LPO (*p* < 0.01), SOD (*p* < 0.0001) and NO (*p* < 0.01) in the heart homogenates (Figure 6). Interestingly, the levels of LPO observed in the catalpol+saline group was statistically lower than that observed in saline group (*p* < 0.0001). The pretreatment with catalpol significantly decreased (*p* < 0.0001–0.01) an increase in these markers of oxidative and nitrosative stress. 

### 3.6. DNA Damage in Heart

**Figure 7:**
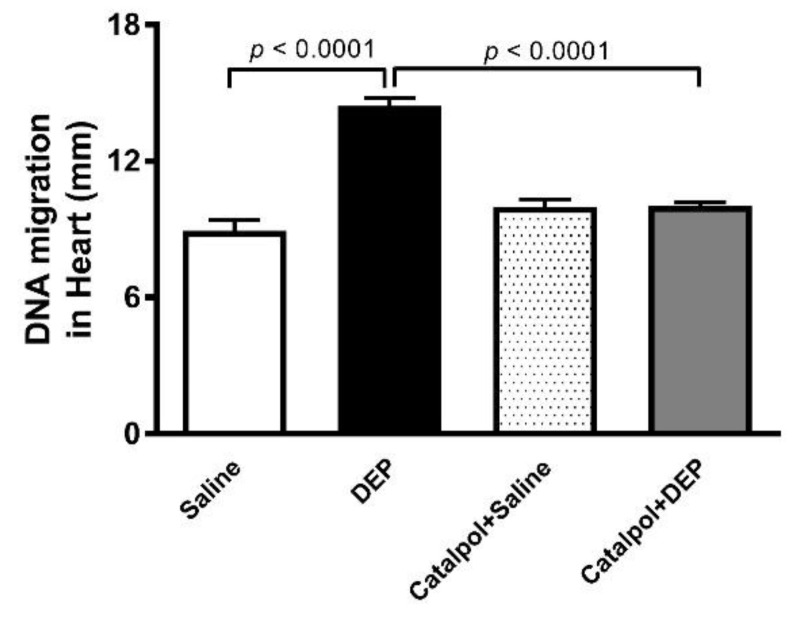
DNA migration (mm) in the heart tissue quantified by Comet assay, 24 h after intratracheal instillation of saline or diesel exhaust particles (DEPs; 30 μg/mouse) with or without catalpol (5 mg/kg) pretreatment. Data are mean ± SEM (*n* = 5). Statistical analysis by one-way analysis of variance followed by Holm-Sidak’s multiple comparisons test.

Compared with the saline group, DEPs exposure induced DNA damage, as evaluated by the comet assay (*p* < 0.0001) (Figure 7). This effect was prevented by the pretreatment with catalpol (*p* < 0.0001). 

### 3.7. Cytochrome C and Cleaved Caspase-3 in Heart Homogenates

**Figure 8:**
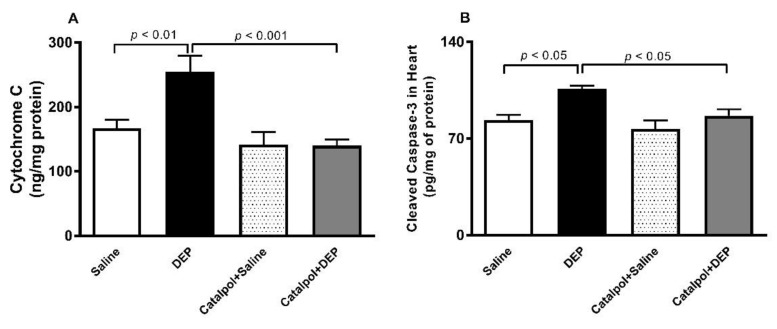
Cytochrome C (**A**) and Cleaved caspase-3 (**B**) levels in heart homogenates, 24 h after intratracheal instillation of saline or diesel exhaust particles (DEPs; 30 μg/mouse) with or without catalpol (5 mg/kg) pretreatment. Data are mean ± SEM (*n* = 6–8). Statistical analysis by one-way analysis of variance followed by Holm-Sidak’s multiple comparisons test.

Figure 8 illustrates that exposure to DEPs induced a significant increase in the levels of cytochrome C (*p* < 0.01) and cleaved caspase-3 (*p* < 0.05), and that pretreatment with catalpol prevented these actions (*p* < 0.001–0.05).

### 3.8. Phopho-NF-κB and Phopho-IκBα in Heart Homogenate

**Figure 9:**
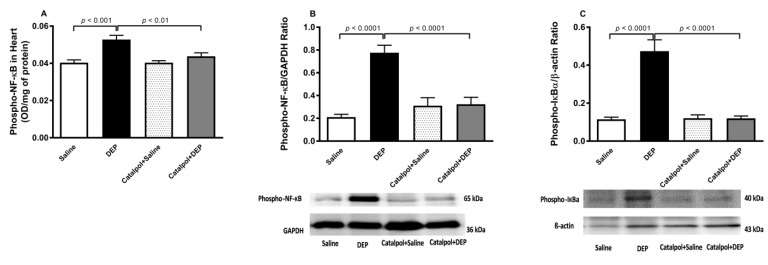
Total levels of phosphorylated nuclear factor-κB (phospho-NF-κB) assessed by either ELISA (**A**) or Western blotting (**B**), and total levels of phospho-NF-κB inhibitor α (IκBα) assessed by Western blotting (**C**) in heart homogenates, 24 h after intratracheal instillation of saline or diesel exhaust particles (DEPs; 30 μg/mouse) with or without catalpol (5 mg/kg) pretreatment. Data are mean ± SEM (*n* = 8). Statistical analysis by one-way analysis of variance, followed by Holm-Sidak’s multiple comparisons test.

DEPs exposure induced a significant elevation in the heart homogenate expression of the total level of phospho-NF-κB, assessed by either ELISA or Wester blotting, and of the phospho-IκBα, assessed by Wester blotting, compared with their respective control groups (saline) (Figure 9). These effects were reversed by pretreatment with catalpol (*p* < 0.01).

## 4. Discussion

In the present study, we demonstrated that catalpol has a salutary impact on DEPs-induced procoagulant effects, heart oxidative and nitrosative stress, inflammation, DNA damage and apoptosis, which occurs, at least partly, through the inhibition of NFκB activation.

The inhalation of particulate air pollution does not only affect the lungs, but it can affect other distant organs including the heart by various mechanisms. These include (1) the passage of biological mediators from the lung into systemic circulation; (2) the translocation of nanoparticles across the alveolar capillary barrier to the blood; and (3) the activation of alveoli sensory receptors, which activates neural afferents that can modify the activity of the autonomic nervous system [3,8]. 

In urban areas, DEPs act as one of the main contributors to particulate air pollution. Accordingly, DEPs have been utilized as a relevant type of pollutant particle, to study the cardiovascular complications associated with particulate air pollution exposure [3]. The dose of DEPs that we utilized in the current work (1 mg/kg or 30 µg/mouse) is similar to those employed previously by us and other authors to study the effects of pulmonary exposure to particulate air pollution [18,32,33]. Pulmonary exposure to DEPs was achieved by i.t. instillation, as it offers more accurate dosing, since mice are nose breathers that filter most of their inhaled particles [15,16,34]. 

Clinical and experimental studies have demonstrated that the inhalation of particulate air pollution increases thrombogenicity both in vivo and in vitro [18,35,36,37]. Our findings showed that exposure to DEPs caused a significant reduction in the thrombotic occlusion time of pial arterioles and venules in vivo, platelet aggregation in vitro and shortened the PT and aPTT, confirming the hypercoagulability action of DEPs. All these effects were significantly reduced following catalpol pretreatment. Catalpol has been reported to attenuate atherosclerotic lesions in a rabbit atherosclerotic model [22]. A recent study has reported that iridoids, such as catalpol, aucubin, and 7-hydroxytomentoside, extracted from the leaves of *Paulownia* Clone, in Vitro 112, exhibit in vitro antiplatelet activity [38]. 

Elevated serum levels of acute-phase proteins such as CRP and fibrinogen, which indicate chronic subclinical inflammation, have been associated with cardiovascular disease [39]. Moreover, an increase in PAI-1 concentrations of plasma has been associated with inflammation and atherosclerosis and has also been recognized as a risk factor for ischemic cardiovascular events [40]. Our data shows that exposure to DEPs caused a significant increase in the plasma concentrations of CRP, fibrinogen and PAI-1, and that catalpol pretreatment prevented these effects, confirming its anti-inflammatory properties. It has been reported that the repeated exposure of mice to DEPs induced an elevation in the plasma concentrations of CRP, fibrinogen and PAI-1, and that treatment with curcumin, the yellow pigment isolated from turmeric, mitigated these effects [41]. The selectins form a family of Ca^2+^-dependent carbohydrate binding proteins, which mediate the first step of white blood cell recruitment in the course of inflammation [42]. Human studies have reported that exposure to particulate air pollution elevates concentrations of soluble P-and E-selectins [43,44]. Here, we showed that plasma concentrations of both P-and E-selectins were significantly augmented by DEPs and that catalpol significantly prevented these effects. The latter finding suggest that catalpol inhibited the increase of endothelial cell and platelet activations, which are caused by DEPs. 

Besides causing systemic inflammation and thrombotic events, it is well established that inhaled particulate air pollutants can adversely affect various organs, particularly the heart. For this reason, we wanted to assess the cardiac impact of DEPs and the potential protective effects of catalpol. Oxidative stress develops as a result of the disproportionate production of reactive oxygen species and the presence of antioxidants or radical scavengers [45]. The excess level of reactive oxygen species assists in oxidizing biomolecules, altering proteins and genes that prompt signalling cascades responsible for the initiation and progression of inflammation [45]. It is well established that catalpol possesses anti-inflammatory and antioxidant properties [10]. However, the possible protective effect of catalpol on particulate air-pollution induced cardiac inflammation and oxidative stress has not been reported on so far. Our findings show that catalpol pretreatment prevented the DEPs-induced increase in cardiac proinflammatory cytokines (TNFα, IL1β and IL-6) and markers of oxidative (LPO and SOD) and nitrosative (NO) stress. It has been shown that catalpol exerts cardioprotective action against ischemia/reperfusion injury by decreasing peroxynitrite formation [11]. 

It is well established that exposure to particulate air pollution can cause DNA oxidation damage which can be triggered by either oxidative stress and/or inflammation [46]. In the current study, using comet assay, we show that i.t. instillation of DEPs induced cardiac DNA damage, and that the pretreatment with catalpol significantly prevented this action. Oxidative DNA damage induced by nanoparticles is capable of prompting mitochondrial injury and apoptosis [47,48]. It is well established that apoptosis can be induced via two pathways [49]. The first is an extrinsic receptor mediated apoptosis in which TNFα triggers apoptosis and the recruitment of caspase family proteins, comprising caspase-8 and caspase-3 [49]. The second intrinsic mitochondrial pathway is induced by the release of cytochrome C from mitochondria, leading to the activation of caspase, caspase-3 [49]. In the present study, we found that pulmonary exposure to DEPs induced a significant increase in the concentration of TNFα in the heart tissue and apoptosis characterized by a significant increase in cytochrome C and cleaved caspase-3. Remarkably, we showed that catalpol pretreatment completely prevented the DEPs-induced elevation in cytochrome C and cleaved caspase-3. Our data corroborate previous in vitro reports that showed that catalpol attenuates cardiomyocyte apoptosis in diabetic cardiomyopathy [10,50,51]. It has been demonstrated that gum Arabic, a prebiotic with anti-oxidant and anti-inflammatory properties, attenuated caspase-3 activation in the heart tissue, triggered by the inhalation of tobacco smoke [52].

In order to further investigate the mechanisms behind the protective effects of catalpol, we evaluated the NF-κB expression in the heart. NF-κB is well-recognized to be involved in the pathophysiology of various inflammatory diseases, particularly those affecting the cardiovascular system such as myocardial infarction and atherosclerosis, as it plays a key role in activating the transcription of inflammatory cytokines, causing inflammation and oxidative stress [53]. The degradation of IκBα induces the nuclear translocation of NF-κB, in which the synthesis of pro-inflammatory cytokines occurs [27]. Our data showed that catalpol treatment prevented the synthesis of pro-inflammatory cytokines (TNFα, IL-1β and IL-6) and NF-κB in the heart. The latter effect can be attributed to the anti-inflammatory activity of catalpol in the heart tissue. Our results are in agreement with a recent in vitro work that reported that catalpol treatment alleviates high glucose-induced apoptosis in mouse cardiomyocytes by inhibiting oxidative stress and suppressing NF-κB activation [50]. 

The limitations of the present work include the fact that we have assessed the protective effect of only one selected dose of catalpol [11,21,22,23] on the acute cardiovascular effects of pulmonary exposure to DEPs. Additional studies are required to assess the impact of various doses of catalpol following subchronic and chronic lung exposure to DEPs, and to include inflammatory cell invasion quantification in the heart and lung by histology. Moreover, in addition to the biochemical techniques used in this study, it would be interesting to confirm our findings using immunohistological studies and to verify the protective effect of catalpol in animal models with pre-existing cardiovascular or respiratory diseases (e.g., hypertension, chronic obstructive pulmonary disease) exposed to DEPs. 

## 5. Conclusions

Taken as a whole, our findings demonstrate that catalpol pretreatment prevented cardiovascular toxicity, induced by acute exposure to DEPs, including procoagulant effects and heart oxidative and nitrosative stress, inflammation, DNA damage and apoptosis through mechanisms involving the inhibition of NFκB activation. Pending further pharmacological and toxicological studies, catalpol may potentially be useful as a cardioprotective agent against the adverse effects of inhaled air pollution. 

## Data Availability

The data presented in this study are available on request from the corresponding author.
